# Supplementary material for: Deep generative AI models analyzing circulating orphan non-coding RNAs enable detection of early-stage lung cancer
Source: Nat Commun. 2024 Nov 21;15:10090. doi: 10.1038/s41467-024-53851-9 (PMC11582319; doi:10.1038/s41467-024-53851-9)
Supplement: Supplementary file 1 — Supplementary Information [file 41467_2024_53851_MOESM1_ESM.pdf]

Supplementary figures and tables

For "Deep generative AI models analyzing circulating orphan non-coding RNAs enable detection of early-stage lung cancer".

Supplementary Table 1: Performance of Orion, ElasticNet, support vector machines (SVM) classifier, and k-NN within the training set (10-fold CV) and held-out validation dataset. For the training set, we chose the cutoff based on the threshold closest to 90% specificity. We applied the same cutoff on the validation set. Values indicate the point estimate and 95% confidence intervals.

Method	AUC	Specificity	Sensitivity	F_1	Dataset
Orion	0.97 (0.96-0.98)	0.90 (0.87-0.93)	0.94 (0.92-0.97)	0.90 (0.88-0.92)	Training set
XGBoost	0.94 (0.93-0.96)	0.90 (0.87-0.93)	0.85 (0.82-0.89)	0.85 (0.82-0.88)	Training set
ElasticNet	0.93 (0.91-0.95)	0.90 (0.88-0.93)	0.81 (0.77-0.85)	0.83 (0.79-0.86)	Training set
KNN	0.80 (0.77-0.83)	0.89 (0.86-0.91)	0.53 (0.48-0.58)	0.62 (0.58-0.67)	Training set
SVM classifier	0.87 (0.84-0.89)	0.90 (0.87-0.93)	0.61 (0.55-0.66)	0.69 (0.64-0.73)	Training set
Orion	0.97 (0.95-0.99)	0.87 (0.81-0.93)	0.93 (0.87-0.98)	0.88 (0.83-0.93)	Validation set
XGBoost	0.95 (0.92-0.97)	0.98 (0.95-1.00)	0.71 (0.61-0.80)	0.81 (0.73-0.87)	Validation set
ElasticNet	0.92 (0.88-0.96)	0.98 (0.95-1.00)	0.44 (0.33-0.54)	0.59 (0.49-0.69)	Validation set
KNN	0.84 (0.78-0.89)	0.98 (0.96-1.00)	0.47 (0.36-0.58)	0.63 (0.53-0.72)	Validation set
SVM classifier	0.89 (0.84-0.93)	0.99 (0.97-1.00)	0.24 (0.15-0.33)	0.38 (0.26-0.50)	Validation set

Supplementary Table 2: Threshold generalizability and sensitivity of Orion and other methods for the validation set at different cutoffs.

Model	Expected specificity	Specificity	Sensitivity	MCC	F1
Orion	0.99	0.98 (0.94–1.00)	0.72 (0.61–0.81)	0.75 (0.66–0.84)	0.82 (0.75–0.89)
Orion	0.95	0.94 (0.89–0.98)	0.89 (0.81–0.95)	0.84 (0.76–0.91)	0.90 (0.85–0.95)
Orion	0.90	0.87 (0.80–0.93)	0.93 (0.85–0.97)	0.79 (0.70–0.87)	0.88 (0.82–0.93)
ElasticNet	0.99	1.00 (0.97–1.00)	0.08 (0.03–0.16)	0.22 (0.13–0.30)	0.15 (0.05–0.25)
ElasticNet	0.95	0.99 (0.96–1.00)	0.29 (0.20–0.40)	0.43 (0.33–0.52)	0.45 (0.33–0.56)
ElasticNet	0.90	0.98 (0.93–1.00)	0.44 (0.33–0.55)	0.52 (0.41–0.62)	0.59 (0.48–0.69)
XGBoost	0.99	1.00 (0.97–1.00)	0.14 (0.08–0.23)	0.30 (0.21–0.39)	0.25 (0.13–0.37)
XGBoost	0.95	0.99 (0.96–1.00)	0.53 (0.42–0.64)	0.62 (0.52–0.71)	0.69 (0.59–0.77)
XGBoost	0.90	0.98 (0.93–1.00)	0.71 (0.60–0.80)	0.73 (0.64–0.82)	0.81 (0.74–0.87)
KNN	0.99	1.00 (0.97–1.00)	0.07 (0.03–0.15)	0.20 (0.12–0.28)	0.13 (0.04–0.22)
KNN	0.95	1.00 (0.97–1.00)	0.07 (0.03–0.15)	0.20 (0.09–0.29)	0.13 (0.03–0.23)
KNN	0.90	0.98 (0.94–1.00)	0.47 (0.36–0.58)	0.56 (0.47–0.65)	0.63 (0.54–0.72)
SVM-classifier	0.99	1.00 (0.97–1.00)	0.07 (0.03–0.15)	0.21 (0.12–0.29)	0.13 (0.05–0.23)
SVM-classifier	0.95	0.99 (0.96–1.00)	0.13 (0.07–0.22)	0.26 (0.15–0.35)	0.23 (0.12–0.33)
SVM-classifier	0.90	0.99 (0.96–1.00)	0.24 (0.15–0.34)	0.37 (0.26–0.47)	0.37 (0.25–0.49)

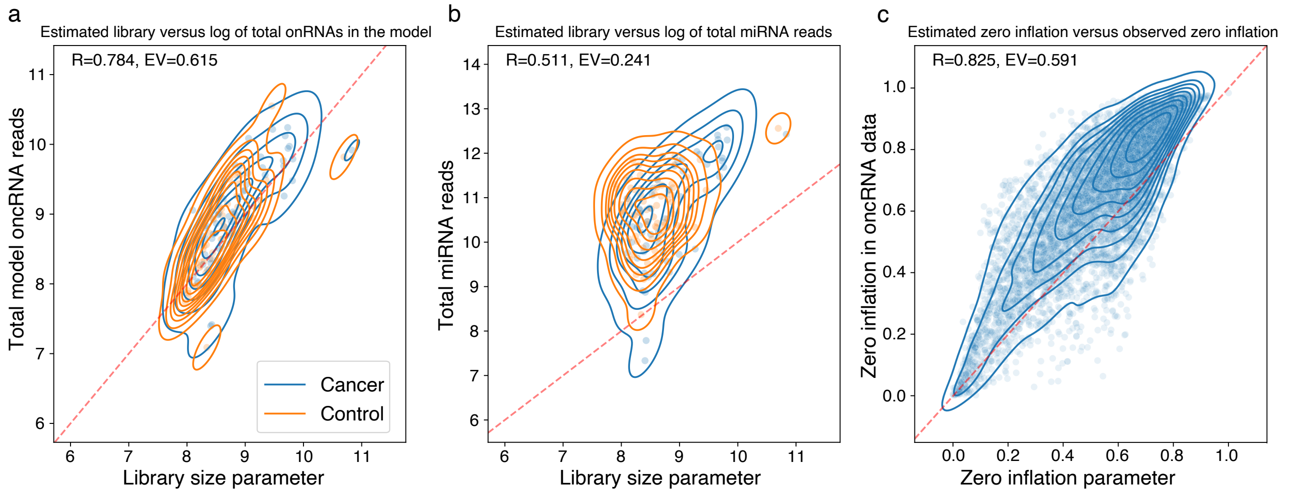

Supplementary Figure 1: Orion properly estimates ZINB parameters. (a) Scatter plot overlaid with kernel density estimates show the estimated library size parameter (x-axis) estimated through the endogenous highly expressed smRNA input, compared to log of the total number of oncRNAs in the input matrix (y-axis). Orange shows control samples, while blue shows cancer samples. Top left annotation indicates Pearson correlation (R) and explained variance (EV). **(b)** Similar to **(a)** but y-axis represents the log of the total number of miRNA reads. **(c)** Estimated zero-inflation of each oncRNA (x-axis) compared to the fraction of the samples expressing that oncRNA (y-axis).

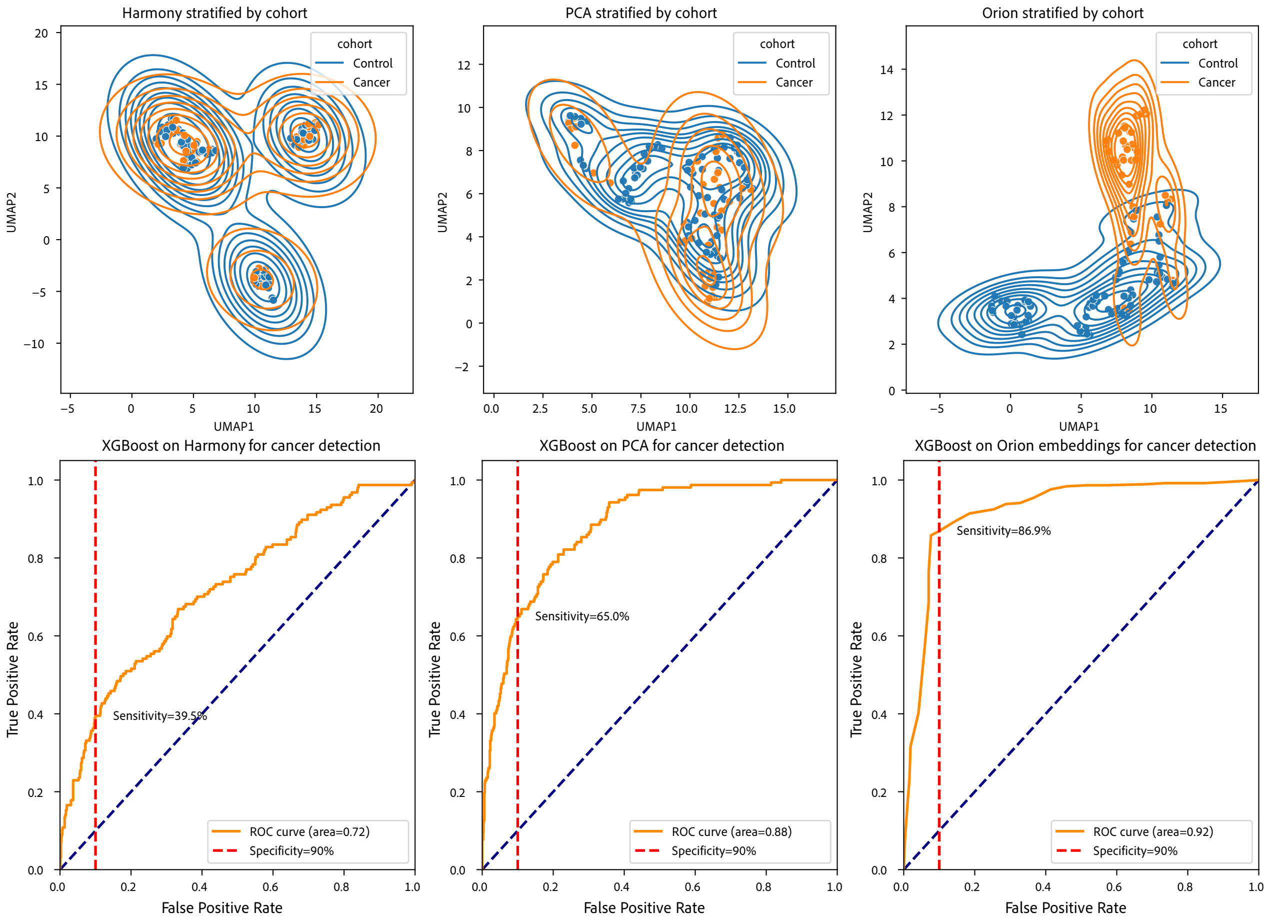

Supplementary Figure 2: Preserving biological signal during batch effect removal. Top panels show the UMAP of embeddings from harmony, PCA, and Orion (tuning set embedding). The bottom panel shows the result of training an xgboost classifier to detect presence of cancer from the top panel embeddings.

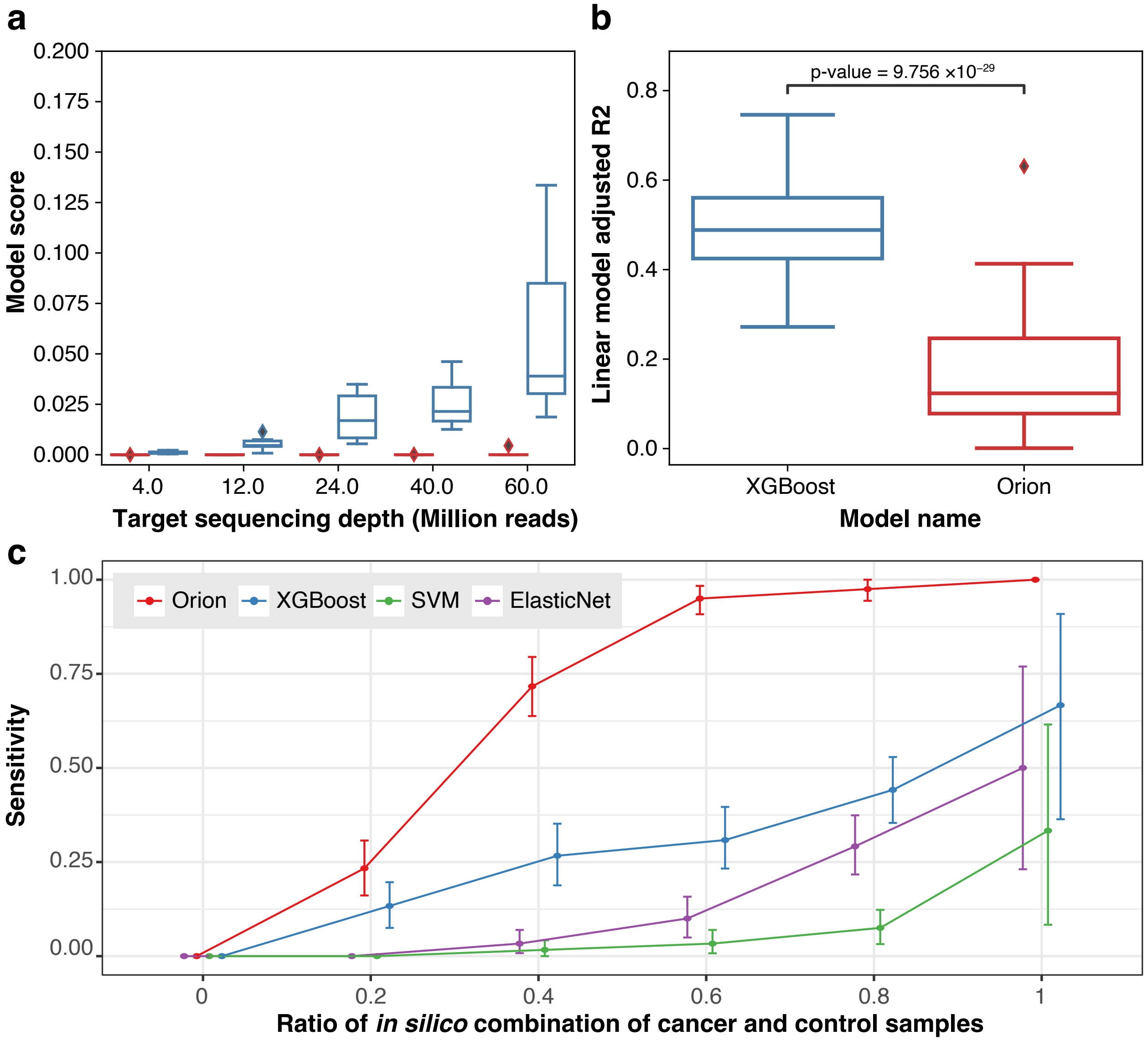

Supplementary Figure 3: Orion robustness and limit of detection. (a) Model score of a pool of the sera from individuals without lung cancer sequenced at different depths. Horizontal axis shows 5 different target sequencing depths ranging from 4 to 60 million reads. Vertical axis shows the model score for Orion (ref) and XGBoost (blue) as boxplots. Horizontal line of boxplot: median. Box range: interquartile range (IQR). Whisker: most extreme value within quartile ± 1.5 IQR. Individual points: outliers beyond a whisker. **(b)** Adjusted R^2 of linear models for association of model scores with target sequencing depth for XGBoost (left; blue) and Orion (right; red). Mann-Whitney U-test two-sided p-value was 9.756×10^{-29} and U-statistic was 9,552. **(c)** Horizontal axis shows the fraction of cancer samples in an *in silico* dilution of cancer and control samples. Vertical axis shows the sensitivity of the assay with respect to the 90% specificity cutoff of each method. Data points correspond to Orion (ref), XGBoost (blue), support vector machine classifier (green), and ElasticNet (purple). Error bars indicate 95% confidence interval.

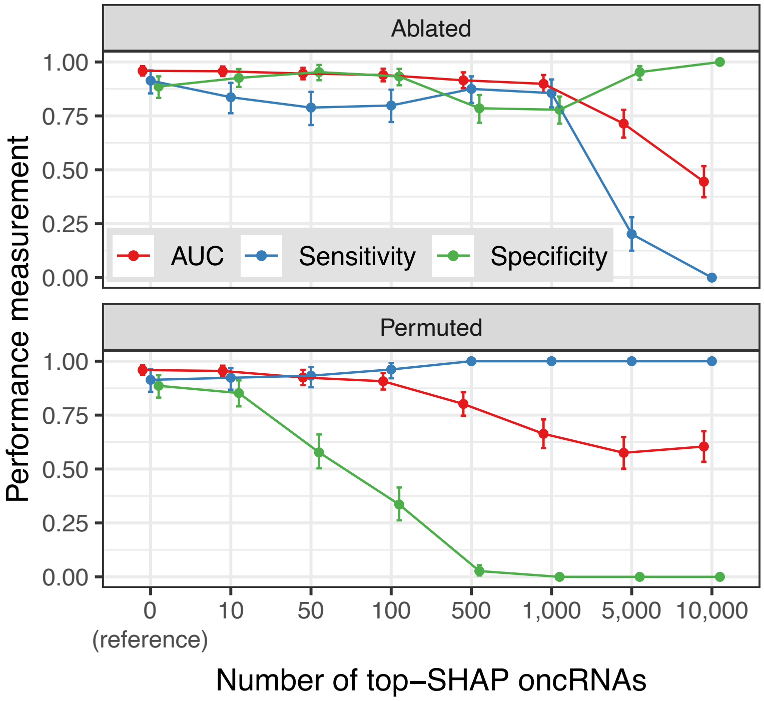

Supplementary Figure 4: Orion tolerance to ablation and permutation of top-SHAP oncRNAs. Horizontal axis shows the number of oncRNAs with highest SHAP values impacted by the experiment. Vertical axis shows model performance as measured by area under ROC (AUC; red), sensitivity (blue), and specificity (green). Top panel shows the impact of setting the top-SHAP oncRNAs to zero among all validation set samples (ablation experiment). Bottom panel shows the impact of permuting top-SHAP oncRNAs among all samples (permutation experiment). Error bars indicate 95% confidence interval.
