# Supplementary material for: Deep generative AI models analyzing circulating orphan non-coding RNAs enable detection of early-stage lung cancer
Source: Nat Commun. 2024 Nov 21;15:10090. doi: 10.1038/s41467-024-53851-9 (PMC11582319; doi:10.1038/s41467-024-53851-9)
Supplement: Supplementary file 2 — Reporting Summary [file 41467_2024_53851_MOESM2_ESM.pdf]

nature portfolio

Corresponding author(s): Fereydoun Hormozdiari, Babak Alipanahi, and Hani Goodarzi

Last updated by author(s): Aug 2, 2024

Reporting Summary

Nature Portfolio wishes to improve the reproducibility of the work that we publish. This form provides structure for consistency and transparency in reporting. For further information on Nature Portfolio policies, see our Editorial Policies and the Editorial Policy Checklist.

Statistics

For all statistical analyses, confirm that the following items are present in the figure legend, table legend, main text, or Methods section.

n/a	Confirmed	
[x]	[ ]	The exact sample size (*n*) for each experimental group/condition, given as a discrete number and unit of measurement
[x]	[ ]	A statement on whether measurements were taken from distinct samples or whether the same sample was measured repeatedly
[ ]	[x]	The statistical test(s) used AND whether they are one- or two-sided<br>*Only common tests should be described solely by name; describe more complex techniques in the Methods section.*
[x]	[ ]	A description of all covariates tested
[x]	[ ]	A description of any assumptions or corrections, such as tests of normality and adjustment for multiple comparisons
[ ]	[x]	A full description of the statistical parameters including central tendency (e.g. means) or other basic estimates (e.g. regression coefficient) AND variation (e.g. standard deviation) or associated estimates of uncertainty (e.g. confidence intervals)
[ ]	[x]	For null hypothesis testing, the test statistic (e.g. *F*, *t*, *r*) with confidence intervals, effect sizes, degrees of freedom and *P* value noted<br>*Give P values as exact values whenever suitable.*
[x]	[ ]	For Bayesian analysis, information on the choice of priors and Markov chain Monte Carlo settings
[x]	[ ]	For hierarchical and complex designs, identification of the appropriate level for tests and full reporting of outcomes
[x]	[ ]	Estimates of effect sizes (e.g. Cohen's *d*, Pearson's *r*), indicating how they were calculated

Our web collection on statistics for biologists contains articles on many of the points above.

Software and code

Policy information about availability of computer code

Data collection	We generated in-house datasets and did not use any software for data collection.
Data analysis	We provided the source code for model architecture, training, and generating predictions. We provided example notebooks for training and reproducing model scores on the validation dataset. These are available at https://github.com/exai-oss/orion.

For manuscripts utilizing custom algorithms or software that are central to the research but not yet described in published literature, software must be made available to editors and reviewers. We strongly encourage code deposition in a community repository (e.g. GitHub). See the Nature Portfolio guidelines for submitting code & software for further information.

Data

Policy information about availability of data

All manuscripts must include a data availability statement. This statement should provide the following information, where applicable:

- Accession codes, unique identifiers, or web links for publicly available datasets
- A description of any restrictions on data availability
- For clinical datasets or third party data, please ensure that the statement adheres to our policy

The raw small RNA-seq data of the non-small lung cancer and adjacent non-cancer tissues of the cancer genome atlas data were obtained through dbGAP (accession phs000178.v11.p8). The count matrices and phenodata of the validation set are available in Zenodo (https://doi.org/10.5281/zenodo.12809652).

Research involving human participants, their data, or biological material

Policy information about studies with human participants or human data. See also policy information about sex, gender (identity/presentation), and sexual orientation and race, ethnicity and racism.

Reporting on sex and gender	We included samples from both female and male individuals in our study design. Complete information is provided as Table 1.
Reporting on race, ethnicity, or other socially relevant groupings	We report summary level information of race and ethnicity in our cohort as Table 1.
Population characteristics	We sourced our samples from two different entities MT Group (Los Angeles, CA) and Indivumed (Hamburg, Germany). To the best of our abilities, we obtained samples with similarities in age, sex, and BMI among the cases and controls.
Recruitment	We sourced our samples from two different entities MT Group (Los Angeles, CA) and Indivumed (Hamburg, Germany).
Ethics oversight	Both entities provided the documentation of IRB approval for the samples used in this study.

Note that full information on the approval of the study protocol must also be provided in the manuscript.

Field-specific reporting

Please select the one below that is the best fit for your research. If you are not sure, read the appropriate sections before making your selection.

[x] Life sciences [ ] Behavioural & social sciences [ ] Ecological, evolutionary & environmental sciences

For a reference copy of the document with all sections, see nature.com/documents/nr-reporting-summary-flat.pdf

Life sciences study design

All studies must disclose on these points even when the disclosure is negative.

Sample size	1050
Data exclusions	- Cancer patient is not treatment naïve at the time of collection<br>- Age ≤ 18<br>- Prior cancer diagnosis (with or without treatment)<br>- Received non-cancer systemic immune modulation therapy within 60 days of collection (e.g., monoclonal antibodies)<br>- History of organ transplantation<br>- Infusion of any blood products within 30 days of collection<br>- Current active COVID-19 infection<br>- Any prior history of cancer therapy (e.g., surgical, radiation, or medical including neoadjuvant treatment)<br>- Current or prior pregnancy within 12 months of collection
Replication	From the beginning, we set aside 20% of the data as a held-out validation set. We observed that our results in the cross-validated training dataset agree with those of the held-out validation set.
Randomization	Whenever possible, including in sample collection, cohort matching, library preparation, sequencing, and dataset splits, we attempted to include covariates including cohort (cancer/control), population characteristics (age/sex/BMI, etc.), and sample source for processing or grouping of the samples.
Blinding	None of the steps included blinding. Given that we benchmark of multiple methods on an identical dataset, no blinding was required for this study.

Reporting for specific materials, systems and methods

We require information from authors about some types of materials, experimental systems and methods used in many studies. Here, indicate whether each material, system or method listed is relevant to your study. If you are not sure if a list item applies to your research, read the appropriate section before selecting a response.

Materials & experimental systems

n/a	Involved in the study	
☒	☐	Antibodies
☒	☐	Eukaryotic cell lines
☒	☐	Palaeontology and archaeology
☒	☐	Animals and other organisms
☐	☒	Clinical data
☒	☐	Dual use research of concern
☒	☐	Plants

Methods

n/a	Involved in the study	
☒	☐	ChIP-seq
☒	☐	Flow cytometry
☒	☐	MRI-based neuroimaging

Clinical data

Policy information about clinical studies
All manuscripts should comply with the ICMJEguidelines for publication of clinical research and a completedCONSORT checklist must be included with all submissions.

Clinical trial registration	Not applicable
Study protocol	Not applicable
Data collection	We sourced our samples from two different entities MT Group (Los Angeles, CA) and Indivumed (Hamburg, Germany). These samples were collected from 2004 to 2023.
Outcomes	All pathological diagnosis including presence of cancer, histology, stage, and T score, were obtained from MT Group (Los Angeles, CA) and Indivumed (Hamburg, Germany)

Plants

Seed stocks	NA
Novel plant genotypes	NA
Authentication	NA
